# Brain Oxygen Perfusion and Oxidative Stress Biomarkers in Fetuses with Congenital Heart Disease—A Retrospective, Case-Control Pilot Study

**DOI:** 10.3390/antiox11020299

**Published:** 2022-01-31

**Authors:** Maria C. Escobar-Diaz, Miriam Pérez-Cruz, Miguel Arráez, Mari-Merce Cascant-Vilaplana, Abel Albiach-Delgado, Julia Kuligowski, Máximo Vento, Narcis Masoller, Maria Dolores Gómez-Roig, Olga Gómez, Joan Sanchez-de-Toledo, Marta Camprubí-Camprubí

**Affiliations:** 1 Pediatric Cardiology Department, Sant Joan de Déu Hospital, 08950 Barcelona, Spain; mariaclara.escobar@sjd.es (M.C.E.-D.); joan.sanchez@sjd.es (J.S.-d.-T.); 2 Sant Joan de Deu Research Institute, 08950 Barcelona, Spain; miguel.arraez@sjd.es (M.A.); lola.gomezroig@sjd.es (M.D.G.-R.); 3 BCNatal-Barcelona Center for Maternal Fetal and Neonatal Medicine, Hospital Clínic, Sant Joan de Déu Hospital, 08950 Barcelona, Spain; masoller@clinic.cat (N.M.); ogomez@clinic.cat (O.G.); 4 Maternal and Child Health and Development Network II (SAMID II), Instituto de Salud Carlos III (ISCIII), Sub-Directorate General for Research Assessment and Promotion and the European Regional Development Fund (ERDF), 28029 Madrid, Spain; 5 Neonatal Research Group, Health Research Institute La Fe, 46026 Valencia, Spain; mari_merce_cascant@iislafe.es (M.-M.C.-V.); abel_albiach@iislafe.es (A.A.-D.); julia.kuligowski@uv.es (J.K.); maximo.vento@uv.es (M.V.); 6 Division of Neonatology, University & Polytechnic Hospital La Fe, 46026 Valencia, Spain; 7 Institut d’Investigacions Biomediques August Pi i Sunyer, Universitat de Barcelona, 08036 Barcelona, Spain; 8 Centre for Biomedical Research on Rare Diseases (CIBER-ER), 08036 Barcelona, Spain; 9 Department of Critical Care Medicine, University of Pittsburgh, Pittsburgh, PA 15213, USA

**Keywords:** congenital heart disease, hypoxia, brain perfusion, reactive oxygen species, ortho-Tyrosine, oxidative stress

## Abstract

Fetuses with congenital heart disease (CHD) have circulatory changes that may lead to predictable blood flow disturbances that may affect normal brain development. Hypoxemia and hypoperfusion may alter the redox balance leading to oxidative stress (OS), that can be assessed measuring stable end-products. OS biomarkers (OSB) were measured in amniotic fluid in fetuses with (*n* = 41) and without CHD (*n* = 44) and analyzed according to aortic flow, expected cyanosis after birth, and a CHD classification derived from this. Birth head circumference (HC) was used as a neurodevelopment biomarker. CHD fetuses had higher levels of ortho-Tyrosine (o-Tyr) than controls (*p* = 0.0003). There were no differences in o-Tyr levels considering aortic flow obstruction (*p* = 0.617). Fetuses with expected extreme cyanosis presented the highest levels of o-Tyr (*p* = 0.003). Among groups of CHD, fetuses without aortic obstruction and extreme cyanosis had the highest levels of o-Tyr (*p* = 0.005). CHD patients had lower HC than controls (*p* = 0.023), without correlation with OSB. Patients with HC < 10th percentile, presented high levels of o-Tyr (*p* = 0.024). Fetuses with CHD showed increased OSB and lower HC when compared to controls, especially those with expected extreme cyanosis. Our results suggest that increased levels of OSB are more influenced by the effect of low oxygenation than by aortic flow obstruction. Future studies with larger sample size are needed to further investigate the role of OSB as an early predictor of neurodevelopmental problems in CHD survivors.

## 1. Introduction

Congenital heart diseases (CHD) are the most common birth defects and a leading cause of morbidity and mortality among infants less than 1 year old [1]. Over the last three decades, advances in prenatal diagnosis, newer surgical techniques and improvements in perioperative management have significantly improved survival with over 85% of patients with CHD reaching adulthood [2]. With an increase in survival rates, long-term morbidity has become the focus of interest in CHD related research [3].

Survivors of CHD are at risk of neurodevelopmental outcome problems. The etiology of this is multifactorial with both genetic and environmental factors playing an important role. There is increasing evidence of structural and functional brain changes during fetal life suggesting that prenatal factors might play an important role in the neurodevelopmental outcome problems of CHD survivors [4,5,6,7]. Recently, Peyvandi and colleagues demonstrated an association between fetuses with smaller brain volume and white matter injury in CHD infants after birth, especially in patients with transposition of the great arteries (TGA) [8]. Moreover, several studies have demonstrated that patients with CHD have smaller head circumference (HC) which is in concordance with delayed brain maturation at birth [4,9,10].

During fetal life, several abnormal patterns of brain oxygen perfusion have been de-scribed in fetuses with CHD: (i) intracardiac shunts with mixed (oxygenated and deox-ygenated) blood; (ii) TGA circulation with hypoxic blood perfusing to the brain, and (iii) other conditions such as hypoplastic left heart syndrome (HLHS) with retrograde perfu-sion to the brain with mixed (oxygenated and deoxygenated) blood from the ductus arteriosus. All these circulatory changes, commonly seen in certain/specific CHD, may lead to predictable blood flow disturbances that may affect normal brain development [11].

Hypoxemia and hypoperfusion may alter the balance between pro-oxidant and an-ti-oxidants, leading to oxidative stress (OS). Excess of reactive oxygen species (ROS) can generate damage to lipids, proteins, and DNA contributing to the pathogenesis of many conditions such as cancer and ischemic stroke complications [12,13]. An increase in ROS after a hypoxic-ischemic event induces apoptosis, inflammation and decreases cell differentiation. Pre-oligodendrocytes, the main dominant cells in brain white matter substance, are also extremely vulnerable to hypoxia-ischemia and can be injured by ROS. Failure of maturation of these pre-oligodendrocytes after acute or chronic hypoxic-ischemic insults could result in impaired myelinization, which, in the brain, translates into a delay in neural maturation [14,15]. All these factors are frequently present in patients with CHD and may play an important role in the neurodevelopment delay reported in this population [16].

The amniotic fluid (AF) compartment reflects the early intrauterine environment of the fetus. AF is essential in fetal development from the beginning of pregnancy, not only by protecting the fetus from potential noxa, but also by allowing a continuous exchange of substances between the fetus and the placenta [17,18]. ROS have been reported to be altered in AF in many scenarios, such as gestational diabetes and intrauterine growth restriction [18,19]. OS in AF can be studied through different biomarkers [18]. The increase of the quotient ortho-Tyrosine (o-Tyr), 3-nitro-Tyrosine (3NO_2_-Tyr), and 3-Chlor-Tyrosine (3Cl-Tyr) relative to their precursors para-Tyrosine (p-Tyr) and Phenyl-alanine (Phe) have proven useful to assess protein oxidation in AF [20]. Recently, a new method for the assessment of damage to proteins in AF has been validated [21].

This study aims were (i) to measure OS biomarkers in a cohort of fetuses with CHD and compare them with controls, (ii) to describe whether a relationship exist between OS and different types of CHD according to oxygen perfusion to the brain (normal, mixed, and low) and aortic flow (normal or obstructed) characteristics, and finally, (iii) to evaluate the relationship between OS biomarkers and the HC at birth.

## 2. Materials and Methods

### 2.1. Patients

This is a retrospective pilot study carried out in a referral tertiary center for CHD (BCNatal, Hospital Sant Joan de Déu and Hospital Clínic Barcelona) including pregnancies from March 2015 to June 2020. The study was conducted according to the guidelines of the Declaration of Helsinki and approved by the Institutional Review Board (or Ethics Committee) (HCB/2015/0365). Informed consent was obtained from all subjects involved in the study.

Cases were selected from fetuses with postnatally confirmed isolated major CHD. The control group included those pregnant women who required an amniocentesis for different clinical indications including extracardiac malformations such as skeletal and facial anomalies, high risk of chromosomal abnormalities and suspicion of congenital infection. Exclusion criteria included those extracardiac malformations with potential impact on OS biomarkers production, mainly structural brain, renal, and thoracic malformations, and confirmed chromosomal abnormalities and/or congenital infections.

Pre-existent maternal hypertension, hypothyroidism, smoking habit, body mass index, and parity were also recorded.

Amniocentesis was performed by a specialist in Fetal Medicine under ultrasound guidance and following the accepted standardized methodology. In most cases, a mean AF volume of 20 mL was obtained, of which 2-mL aliquots were stored at –80 °C until the biochemical analysis.

Postnatal weight and HC were recorded at birth. Raw data were transformed into z-scores in reference to intergrowth-21 charts curves for comparison with the population of the same gestational age at birth [22].

### 2.2. CHD Classification

CHD lesions were classified into different groups based on the presence of antegrade aortic flow (obstructed vs non-obstructed) and the expected level of hypoxemia after birth (no-, mild-, extreme-cyanosis) based on a previously described classification [23].

**Figure 1:**
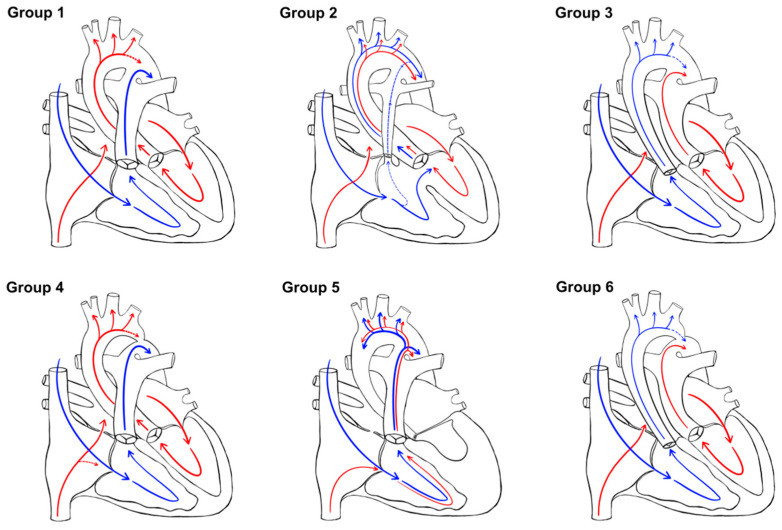
Congenital heart disease classification based on expected oxygen delivery to the brain (normal, mixed, and low), and antegrade aortic flow (obstructed or not). Group 1: normal oxygen with normal aortic flow (no obstruction) (CHD without significant intracardiac shunt); Group 2: mixed oxygen with normal aortic flow (no obstruction) (CHD with intracardiac shunt); Group 3: low oxygen with normal aortic flow (no obstruction) (TGA physiology without significant intracardiac shunt); Group 4: normal oxygen with obstructed aortic flow (CHD without significant intracardiac shunt); Group 5: mixed oxygen with obstructed aortic flow (CHD with intracardiac shunt, mainly HLHS); Group 6: low oxygen with obstructed perfusion (TGA physiology without significant intracardiac shunt). CHD: congenital heart disease, TGA: transposition of the great arteries, HLHS: hypoplastic left heart syndrome.

Finally, similarly as previously published by Donofrio and colleagues [11], CHD were clustered based on the two mentioned criteria: expected oxygen delivery to the brain (normal, mixed, and low), and antegrade aortic flow (obstructed or not) as depicted in Figure 1.

- Group 1: normal oxygen with normal aortic flow (no obstruction) (CHD without significant intracardiac shunt)
- Group 2: mixed oxygen with normal aortic flow (no obstruction) (CHD with intracardiac shunt)
- Group 3: low oxygen with normal aortic flow (no obstruction) (TGA physiology without significant intracardiac shunt)
- Group 4: normal oxygen with obstructed aortic flow (CHD without significant intracardiac shunt)
- Group 5: mixed oxygen with obstructed aortic flow (CHD with intracardiac shunt, mainly HLHS)
- Group 6: low oxygen with obstructed perfusion (TGA physiology without significant intracardiac shunt).

### 2.3. Standards

Standards of o-Tyr, p-Tyr, Phe, 3NO_2_-Tyr, 3Cl-Tyr, 8OHdG and 2dG (>96% *w*/*w* purity) were from Sigma-Aldrich (St. Louis, MO, USA). Internal standards (ISs) p-Tyr-D_2_, 2dG-^13^C^15^N_2_, and 8OHdG-^13^C^15^N_2_ were acquired from Cambridge Isotope Laboratories and Phe-D_5_ from CDN Isotopes (Pointe-Claire, Canada).

Individual stock solutions of o-Tyr (2 mM), 3NO_2_-Tyr (2 mM), 3Cl-Tyr (2 mM), 8OHdG (2 mM), 2dG (2 mM), 2dG-^13^C^15^N_2_ (5 mM), 8OHdG-^13^C^15^N_2_ (5 mM), Phe-D_5_ (10 mM) and Phe (75 mM) were prepared and dissolved as previously described [21]. Once prepared, solutions were stored at −20 °C. Multi-component working solutions were prepared and kept at −20 °C. Standard solutions were prepared by serial dilution of the working solutions. The preparation of the standards and reagents that we used were described in detail by Cascant-Vilaplana and colleagues [21].

### 2.4. AF Sample Preparation and Analysis

AF samples were homogenized and centrifuged (5 min, 10,000 g, 4 °C). 250 μL of phosphoric acid solution (5%, *w*/*v*) containing Phe-D_5_, 8OHdG-^13^C^15^N_2_, 2dG-^13^C^15^N_2_ and p-Tyr-D_2_ at 0.1 μM as ISs were mixed with 250 µL of AF supernatant. ISOLUTE^®^-96 ENV+ (96 well, 40 mg) plates from Biotage (Uppsala, Sweden) were used for solid phase extraction. Plate wells were conditioned with 1 mL of CH_3_OH and 1 mL of H_2_O. Samples were loaded, cartridges were washed (2 × 300 µL of H_2_O), and eluted using NaOH (150 µL, 0.1 M) and CH_3_CN (2 × 100 µL aand 50 µL). Recovered extracts were evaporated and dissolved in 50 µL of 0.1% *v*/*v* HCOOH. In addition, a 1:400 diluted sample extract was prepared in 0.1% *v*/*v* HCOOH. Redissolved and diluted extracts were analyzed by Ultra Performance Liquid Chromatography—tandem Mass Spectrometry (UPLC-MS/MS). Oxidized compounds (i.e., o-Tyr, 3NO2-Tyr, 3Cl-Tyr, 8OhdG, and 2dG) were determined in sample extracts, while diluted samples were employed to determine precursors (i.e., p-Tyr and Phe). Sample preparations and their analysis using UPLC-MS/MS method were thoroughly explained by Cascant-Vilaplana and colleagues [21].

### 2.5. Statistical Analysis

According to the distribution of the data, quantitative variables were expressed as mean and standard deviation of mean (SD) or as median and interquartile range (IQR). For the comparison of both global study groups, the parametric independent-sample t test and nonparametric Mann–Whitney U test were used for continuous variables that did or did not meet the assumption of normality, respectively. When the comparison included different CHD classifications, ANOVA test or Kruskal–Wallis test, also depending on the normality of the variable, were used. Post-hoc analysis was performed using Bonferroni. Correlations were performed using the Spearman correlation coefficient, rho. Confounding factors were analyzed using linear regression. Statistical significance was considered when *p* < 0.05. SPSS version 25 (IBM, Armonk, NY, USA) and STATA v13 package were used for the statistical analyzes.

## 3. Results

### 3.1. Population of the Study

**Figure 2:**
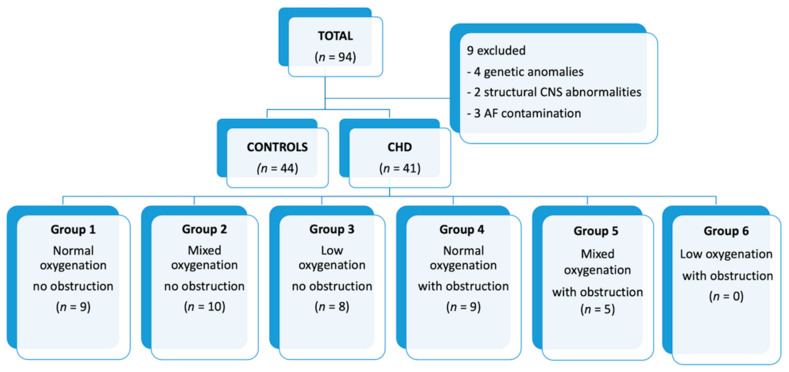
Distribution of patients. CHD: congenital heart disease; CNS: central nervous system; AF: amniotic fluid.

A total of 94 AF samples were collected, of which 85 were analyzed, including 41 CHD and 44 controls. The distribution of the patients is shown in the flow-chart (Figure 2).

Nine AF samples were excluded due to genetic anomalies, structural CNS abnormalities associated with the CHD, and AF contamination. Demographic characteristics of our population are described in Table 1. 

Table 2 shows the diagnoses of CHD by groups according to the classification previously described.

**Figure 3:**
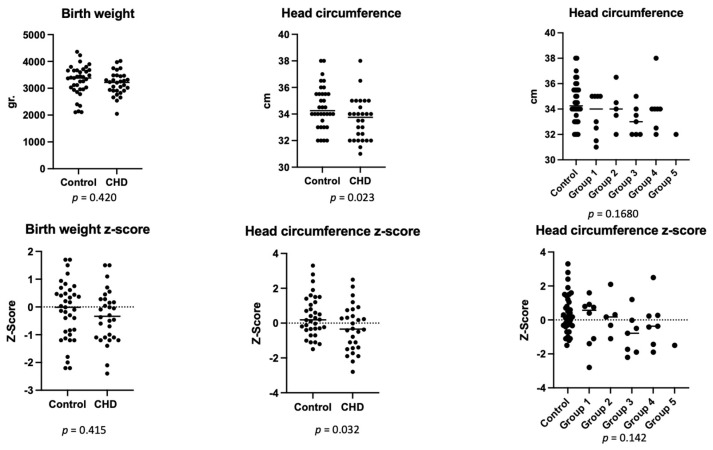
Neonatal biometrics.

There were 72 liveborns (29 cases and 43 controls). Twelve families from the CHD group elected for termination of pregnancy (4 with HLHS, 4 pulmonary atresia, 3 complex CHD, 1 severe aortic coarctation). Gestational age and birthweight were similar between groups (*p* = 0.9, *p* = 0.420). As expected, CHD patients had a smaller HC at birth (*p* = 0.023), with a lower z-score (*p* = 0.036), a finding that seems to correlate with the type of CHD, with group 3 showing the most important reduction (Figure 3).

### 3.2. Amniotic Fluid Analysis

Concentration of o-Tyr in AF was significantly higher in CHD pregnancies compared to controls (3.27 ± 0.76 ug/L vs. 2.68 ± 0.64 ug/L, *p* = 0.0003). Main results for the different OS biomarkers are shown in Table 3.

OS parameters were analyzed considering the main comorbidities and pre-existing conditions of pregnancy. Levels of NO2Tyr and the relation 3NO2-Tyr/p-Tyr were increased in those pregnancies with preeclampsia (*p* = 0.001; *p* = 0.029). There were no differences in OS biomarkers considering maternal hypothyroidism and smoking habit. There was a tendency of increased o-Tyr levels in pregnancies with diabetes (*p* = 0.061).

**Figure 4:**
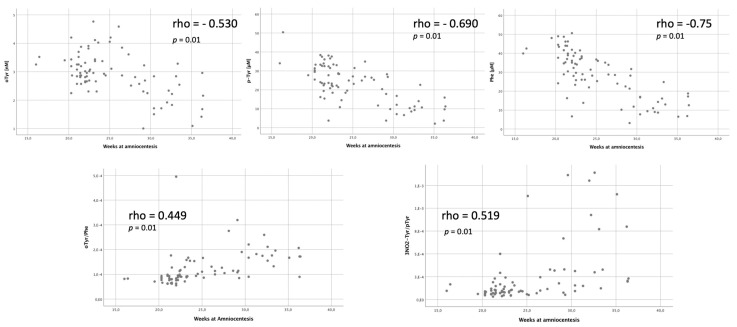
Correlation of OS Biomarkers and gestational age at the time of amniocentesis. o-Tyr: ortho-Tyrosine, p-Tyr para-Tyrosine, Phe Phenylalanine, 3NO_2_-Tyr: 3-nitro-Tyrosine.

Considering gestational age at the time of amniocentesis, there was a negative correlation with the levels of o-Tyr, p-Tyr and Phe (rho = −0.530; *p* = 0.01; rho =−0.690; *p* = 0.01; rho = −0.75, *p* = 0.01) and a positive correlation with o-Tyr/Phe and 3NO_2_-Tyr/p-Tyr (rho = 0.449, *p* = 0.01; rho = 0.519, *p* = 0.01) (Figure 4). Despite these findings, the comparative study of these potential confounders did not show differences between CHD pregnancies and controls (Table 1).

OS biomarkers were analyzed according to (1) aortic flow obstruction, (2) expected cyanosis after birth, and (3) our proposed CHD classification.

When analyzing the cohort considering aortic flow obstruction, no differences in o-Tyr levels were detected between obstructed and non-obstructed CHD (*p* = 0.912).

**Figure 5:**
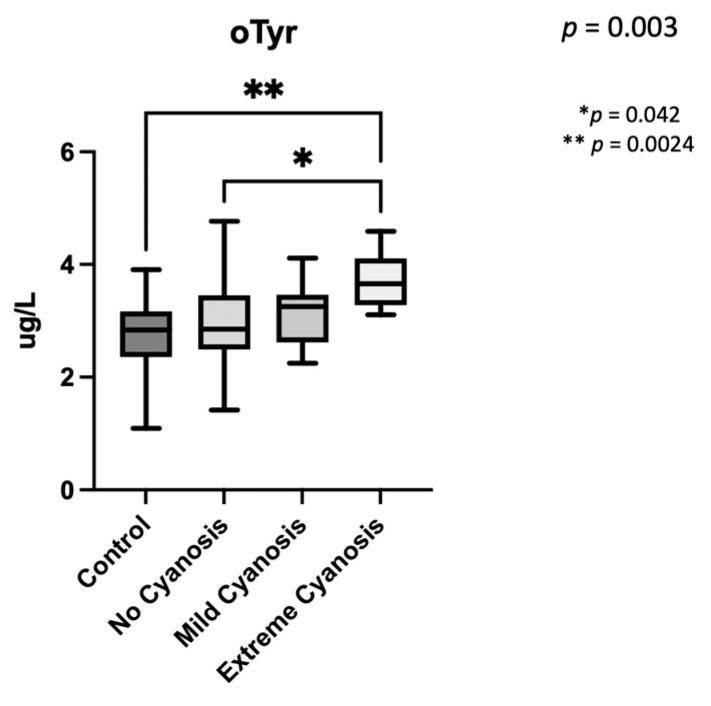
Ortho-Tyrosine levels depending on expected cyanosis after birth. The global *p* of the analysis was *p* = 0.003. (*) remarks the *p* obtained in the post-hoc analysis when controls and extreme-cyanosis patients were compared, and (**) is used to indicate *p*-value for the statistical differences between extreme-cyanosis and no cyanosis. o-Tyr: ortho-Tyrosine.

Regarding the theoretically cyanosis after birth, those cases with expected postnatal extreme-cyanosis (TGA) had the highest levels of o-Tyr, being, in the post-hoc analysis, different when compared to the control group (*p* = 0.0024), and to the no-cyanosis group (*p* = 0.042) (Figure 5).

**Figure 6:**
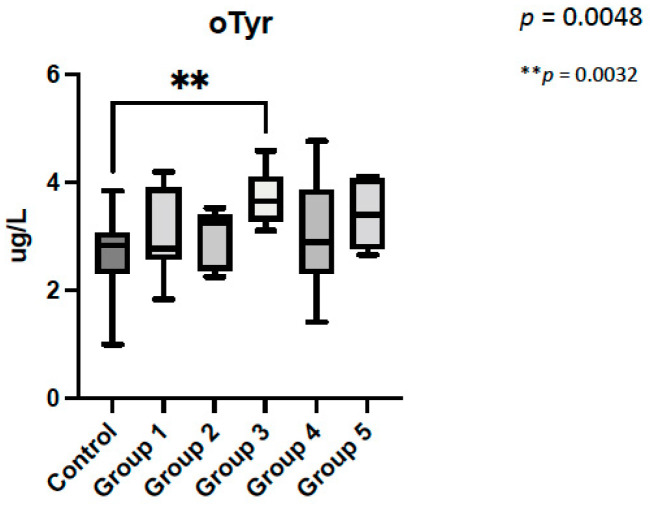
Ortho-Tyrosine levels depending on our congenital heart disease classification. The global *p* of the analysis was *p* = 0.0048. ** *p* = 0.0032, remarks the *p* obtained in the post-hoc analysis when controls and group 3 were compared. o-Tyr: ortho-Tyrosine.

When comparing between groups according to our proposed classification, the highest o-Tyr levels were found in group 3 (low oxygenation and non-obstructed aortic flow CHD) (*p* = 0.005) (Figure 6).

Finally, and in relation to the cephalic neonatal biometrics, no correlation between HC or z-score HC and OS biomarkers was found (*p* = 0.22) but those patients with a HC under the 10th percentile (p10), presented increased levels of o-Tyr (*p* = 0.024). Among CHD patients, up to 10% presented a HC under p10, while in the control group only 2% were in p10 (*p* = 0.052).

## 4. Discussion

Our data show, for the first time, that fetuses with CHD present significantly increased o-Tyr levels in AF compared to control group. Interestingly, low oxygenated-CHD (group 3) presented the highest levels of protein oxidation byproducts. Newborns with a HC under the 10th percentile showed significantly increased AF o-Tyr levels. Moreover, our results suggest that increased levels of OS biomarkers are more influenced by the effect of low cerebral oxygenation rather than by the presence of aortic flow obstruction.

### 4.1. Fetal Brain Perfusion and Oxidative Stress

For decades, the relationship between expected brain perfusion in CHD and brain development has been thoroughly studied [9,10,24,25,26,27].

During their life, patients with CHD are subjected to several periods of hypoxia/hypoperfusion, including fetal life, transition from fetal to neonatal period and moments of hemodynamic instability in the perioperative periods [28]. The generation of OS during episodes of hypoxia-hyperoxia and ischemia-reperfusion has been associated with the pathophysiology of different conditions [14]. Moreover, the association of OS specifically with brain maturation has also been reported in other neonatal diseases such as prematurity and hypoxic-ischemic encephalopathy [29,30]. The increase of ROS after a hypoxic-ischemic event has been related to apoptosis and decreased cell differentiation, which, in the brain, translates into a delay in neural maturation [14]. There is mounting evidence that brain injury or altered development of white matter, in CHD patients, may be present already during fetal life [16].

### 4.2. Fetal Brain Perfusion and Oxidative Stress in CHD

Fetal brain blood flow is influenced by multiple factors, including the structure of the heart and the impedance of the distal vascular beds. The circulatory alterations that accompany specific anatomic CHD led to different patterns of blood flow disturbance that may affect normal brain development also altering the imbalance of the OS status [11]. In our cohort, fetuses with CHD presented increased levels of o-Tyr when compared to the control group. It has been reported that when hydroxyl radicals oxidize the benzyl ring of phenylalanine, abnormal levels of tyrosine isomers such as o-Tyr are produced [31]. Remarkably, these protein oxidation biomarkers have been also previously associated to other pregnancy conditions, most of them related to chronic fetal hypoxia [32].

### 4.3. Oxidative Stress Biomarkers Are Increased in CHD with Low Brain Oxygenation

When cases were analyzed considering cyanosis (no cyanosis, mild, extreme), those with expected extreme cyanosis (TGA) were the ones with the highest levels of o-Tyr and its precursors. Recently, our group demonstrated that after cardiac surgery, there is an increase in OS, especially in the early neonatal period and in patients with TGA. These findings support the hypothesis that the changes in oxygenation and perfusion play an important role in redox balance. Similarly, to what we have detected in the present study, different pre-surgical OS profiles were found depending on the degree of expected cyanosis [23].

Contrary to what we initially expected, OS biomarkers did not differ based on the presence/absence of aortic flow obstruction. These results suggest that increased levels of OS biomarkers may perhaps be more related to the effect of low cerebral oxygenation than the degree of aortic flow obstruction. This finding was evident even in patients with HLHS, classically considered one of the CHD with higher risk of neurodevelopmental delay [31]. In this group of patients, OS biomarker levels were not different, maybe due to the small sample size. Mechanisms of cerebral protection, such as the brain sparing effect described by Donofrio and colleagues [11], could provide an explanation.

According with our CHD classification, those patients included in the group 3 (low oxygenation with normal aortic flow) were the ones with the highest levels of o-Tyr, being different even from those patients included in group 5 (mixed oxygenation and aortic flow obstruction).

Classically, patients with TGA and HLHS have been described as the ones with the worst neurodevelopment outcome [33,34,35]. Recently, some authors have described that a decrease in total brain volume is associated with increased risk of postnatal white matter injury especially in TGA, but not in HLHS patients [8]. In the same line, Everwijn and colleagues reported that only TGA fetuses present a delay in brain maturation. In their study, fetuses with HLHS did not show significantly less mature brains [24]. All these results could support the differences that we have found between TGA and HLHS patients in our cohort.

### 4.4. Head Circumference Is Lower in CHD Patients

Indirect neurodevelopmental biomarkers such as HC were lower in CDH patients despite using z-score that corrects for gestational age at birth. HC was also lower in low oxygenation groups (group 3) and with any degree of flow obstruction (groups 4 and 5). However, differences were nonsignificant.

Our results agree with those found by Jansen and colleagues, who described a decline in HC growth in fetuses with CHD irrespective of aortic flow [10]. In addition, in a nationwide study in Denmark, patients with CHD also had smaller HC at birth. Interestingly, patients with TGA presented smaller HC in relation to birthweight [36]. Going further, a recent study published by our group, demonstrated that those CHD fetuses with an expected lower brain oxygen supply had a smaller corpus callosum [27]. All these data support the hypothesis that circulatory changes in CHD could disrupt oxygen and nutrient supply to the brain with a significant impact on brain maturation and growth. In our cohort, there was no correlation between HC or z-score HC and OS biomarkers but those patients with a HC under p10, presented increased levels of o-Tyr.

### 4.5. Strengths and Limitations of the Study

To our knowledge, this is the first study to report the relation between AF OS biomarkers and CHD in pregnancy. Limitations of the study include first being a retrospective study and therefore the neurodevelopmental outcome of these patients was not prospectively examined and only indirect measures, such as HC, could be analyzed. Moreover, the sample size is small, especially in the main sub-groups of CHD such as TGA and HLHS, decreasing the power of the results. We are conducting a prospective study with a larger cohort of fetuses to analyze type-specific CHD on OS biomarker profiles and their relationship with neurodevelopment in these groups of patients.

## 5. Conclusions

Fetuses with CHD showed increased OS biomarkers levels and lower HC when compared to controls. These differences were more pronounced in patients with CHD with low brain oxygenation. Moreover, our results suggest that increased levels of OS biomarkers are more influenced by the effect of low cerebral oxygenation rather than by the presence of aortic flow obstruction. Future studies encompassing a larger number of cases are needed to further investigate the role of OS biomarkers as an early predictor of neurodevelopmental outcome problems in CHD survivors.

## Figures and Tables

**Table 1:** Baseline and perinatal characteristics of the study population.

Clinical Characteristics	Control Group (*n* = 44)	CHD Group (*n* = 41)	*p* Value
Age (years)	33.1 ± 5	34.2 ± 5	0.36
Amniocentesis weeks	26.2 ± 5	24.20 ± 5	0.088
Parity	0 [0–1]	1 [0–1]	0.94
Body mass index (kg/m^2^)	25 ± 7	27 ± 3	0.61
Hypothyroidism %	0	20	0.30
Preeclampsia %	4.3	2.8	0.86
Gestational diabetes %	5.7	8.1	0.50
Smoking habit %	12.8	9.5	0.96
Gestational age at birth (weeks)	39.4 ± 1.4	39.4 ± 0.9	0.91
Neonatal birth-weight (gr)	3280 ± 559	3178 ± 436	0.42
Birth-weight percentile	51 ± 28	39 ± 26	0.12
Head circumference at birth (cm)	34.5 ± 1.6	33.53 ± 1.5	0.023
Head circumference percentile	59 ± 30	44 ± 31	0.077
Head circumference z-score	0.38 ± 1.17	−0.29 ± 1.3	0.044
Amniocentesis indication	Club foot: 26% Cleft palate:10% Vascular ring: 12% Short long bones: 5% Suspected CNS alterations: 23% Suspected infection: 3% Others: 21%	CHD Classification Group 1: 22% (*n* = 9) Group 2: 24% (*n* = 10) Group 3: 20% (*n* = 8) Group 4: 22% (*n* = 9) Group 5: 12% (*n* = 5) Group 6: 0	

CHD: congenital heart disease; CNS: central nervous system.

**Table 2:** Congenital heart disease diagnoses by groups.

CHD Classification	Number of Patients
Group 1 Moderate sized ventricular septal defect Tetralogy of Fallot without pulmonary stenosis L-TGA without other malformations Mild pulmonary stenosis	(*n* = 9) 4 3 1 1
Group 2 DORV with pulmonary atresia DORV + TGA Tetralogy of Fallot with pulmonary stenosis/atresia CHD with single ventricle physiology Pulmonary atresia with VSD	(*n* = 10) 1 2 2 3 2
Group 3 TGA with intact ventricular septum TGA with VSD	(*n* = 8) 4 4
Group 4 Moderate aortic stenosis Shone complex Aortic coartaction Aortic coartaction with VSD Truncus arteriosus and interrupted aortic arch	(*n* = 9) 3 1 2 2 1
Group 5 HLHS Unbalanced AVSD with hypoplastic left ventricle	(*n* = 5) 4 1
Group 6 TGA + aortic coarctation	0

TGA: transposition of great arteries; DORV: double outlet right ventricle; CHD congenital heart disease; VSD: ventricular septal defect; HLHS: hypoplastic left heart syndrome.

**Table 3:** Oxidative stress biomarkers in amniotic fluid.

	Control	CHD	*p* Value
o-Tyr [nm]	2.68 ± 0.64	3.27 ± 0.76	0.0003
No2-Tyr [nm]	3.71 ± 3.19	3.52 ± 4.4	0.82
p-Tyr [µm]	20.97 ± 9.8	25.42 ± 9.8	0.045
Phe [µm]	26.62 ± 13	31.36 ± 11.30	0.084
o-Tyr/Phe	0.000126 ± 0.0000618	0.0001241 ± 0.0000755	0.89
3No2-Tyr/p-Tyr	0.0002648 ± 0.000335	0.0001939 ± 0.0002921	0.31

o-Tyr: ortho-Tyrosine, p-Tyr para-Tyrosine, Phe Phenylalanine, 3NO2-Tyr: 3-nitro-Tyrosine.

## Data Availability

Main data is contained within the article. More specific data are available from the corresponding author on reasonable request.
